# Changes in the N-glycosylation of porcine immune globulin G during postnatal development

**DOI:** 10.3389/fimmu.2024.1361240

**Published:** 2024-04-18

**Authors:** Kristina Zlatina, Lisa Isernhagen, Christina E. Galuska, Eduard Murani, Sebastian P. Galuska

**Affiliations:** Research Institute for Farm Animal Biology (FBN), Dummerstorf, Germany

**Keywords:** porcine IgG, N-glycan, antibody, glycosylation, pig, ontogenesis

## Abstract

N-glycosylation influences the effectiveness of immune globulin G (IgG) and thus the immunological downstream responses of immune cells. This impact arises from the presence of N-glycans within the Fc region, which not only alters the conformation of IgG but also influences its steric hindrance. Consequently, these modifications affect the interaction between IgG and its binding partners within the immune system. Moreover, this posttranslational modification vary according to the physiological condition of each individual. In this study, we examined the N-glycosylation of IgG in pigs from birth to five months of age. Our analysis identified a total of 48 distinct N-glycan structures. Remarkably, we observed defined changes in the composition of these N-glycans during postnatal development. The presence of agalactosylated and sialylated structures increases in relation to the number of N-glycans terminated by galactose residues during the first months of life. This shift may indicate a transition from passively transferred antibodies from the colostrum of the sow to the active production of endogenous IgG by the pig’s own immune system.

## Graphical Abstract

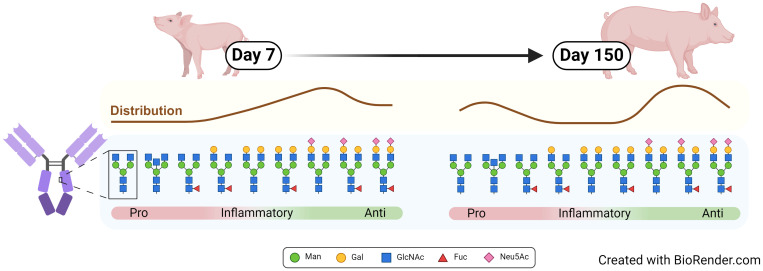

## 1 Introduction

**Figure 1:**
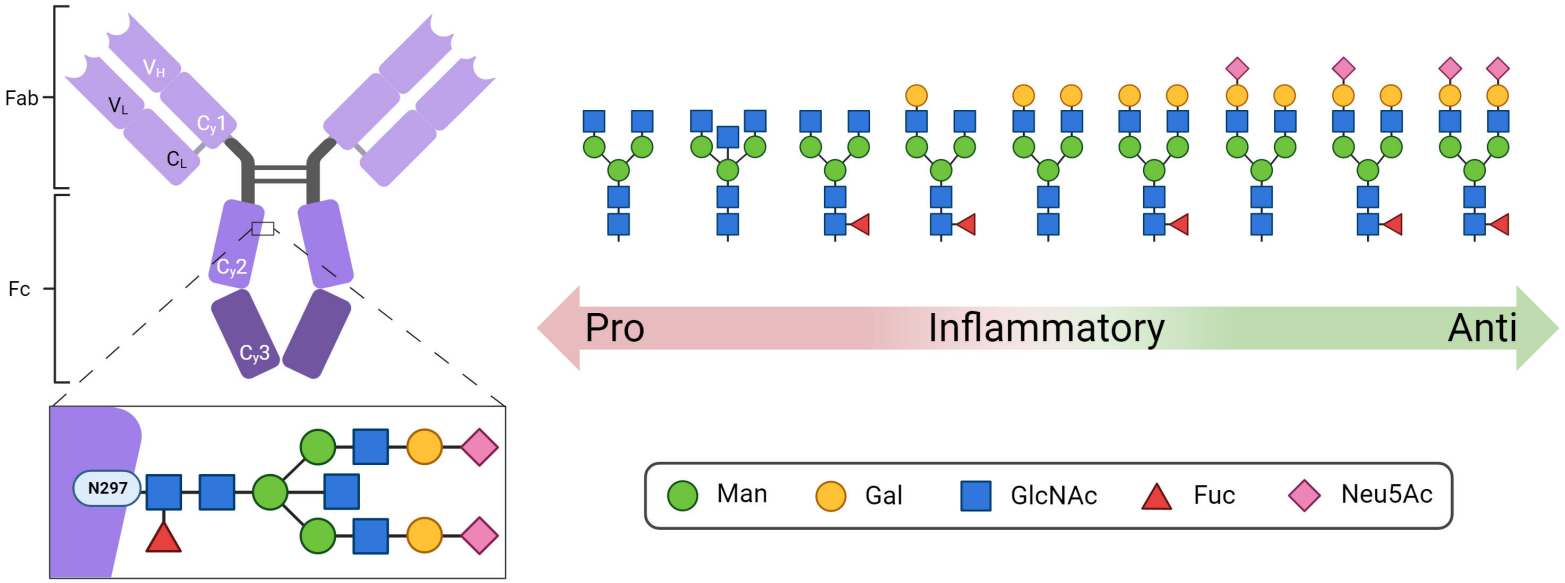
Structure of IgG with the conserved N-glycosylation site at asparagine (N) 297 in the Fc domain. Shown are representative N-glycan structures of human IgG grouped according to their potential to trigger pro- or anti-inflammatory effector functions of the immune system. Man, mannose; Gal, galactose; GlcNAc, N-acetylglucosamine; Fuc, fucose; Neu5Ac, N-acetylneuraminic acid. Created with BioRender.com.

Immune globulins (Ig) play an essential role in the orchestration of the adaptive immune system. The most abundant immune globulin in the bloodstream is immune globulin G (IgG), which is glycosylated at a conserved sequence within the Fc region at asparagine (Asn) 297 (**Figure 1**) (1). Since N-glycans at Asn297 are located close to the hinge region, their composition directly influences the conformation of the complete Fc region and thus the interaction with Fc receptors and complement factors. Accordingly, the N-glycans at Asn297 are key factors that modulate the response of the immune system (1). This includes pro-inflammatory responses such as antibody-dependent cellular phagocytosis, antibody-dependent cell-mediated cytotoxicity (2–7), complement-dependent cytotoxicity (8, 9), and anti-inflammatory responses (10, 11). Remarkably, IgGs whose Fc N-glycans are closest to paucimannose, the N-glycan core structure including two N-acetylglucosamine (GlcNAc) and three mannose (Man) residues, induce pro-inflammatory responses (**Figure 1**), whereas IgGs with elongated complex N-glycans containing additional galactose (Gal), fucose (Fuc), and N-acetylneuraminic acid (Neu5Ac) residues lead to greater anti-inflammatory effects (12, 13). In addition, around 15-25% of human IgGs also have Fab-N glycans (14).

The glycosylation pattern of IgG is influenced by different factors, including sex hormones (15, 16), age (17), lifestyle (18), and immunological status (13, 19). Pathophysiological processes, such as infections (20), endogenous inflammation (21), and autoimmune diseases (22), also modulate and alter the N-glycosylation status of IgGs. In general, the N-glycosylation of IgG occurs in the endoplasmic reticulum and Golgi apparatus. The expression levels of glycosyltransferases and glycosidases, the availability of monosaccharides, the Golgi topology and pH, and the translation, folding and degradation of proteins, in addition to the transport mechanisms, are additional variable factors that influence the glycosylation patterns at Asn297 and thus the IgG conformation and subsequent effector functions (17). However, the interplay of all these variables is still unclear.

While extensive research has been conducted on the glycosylation of human IgG, our understanding in the context of pigs is limited. Several crucial aspects of glycosylation in pigs have not been identified. For example, how glycosylation changes with age and under altered physiological conditions. This includes the first passive immunization of the offspring with maternal Igs. Intriguingly, the anatomical structure of the thick epitheliochorial placenta in pigs means that Igs cannot be transferred via the placenta as they are in humans. Piglets receive their first immunization through milk. In particular, the first milk, called colostrum, is important since the gut barrier of piglets is permeable during the first days, and components of the milk, such as IgG, can be transferred into the blood circulation. In addition to IgG, milk also contains IgA and IgM, whereby IgA is especially important for the immune barrier of mucosal surfaces such as gastrointestinal and respiratory tracts. However, in contrast to IgG, the impact of the N-glycans on the activity of IgA and IgM has been little studied to date. All these milk Igs are the first passive immunizations and are essential for piglet health and survival. For this reason, dams are vaccinated during pregnancy to reduce piglet mortality. Thus, research on porcine antibodies and their glycosylation could yield valuable insights into the immune system, potentially enhancing our understanding of vaccine development and ultimately improving the health of sows and piglets. By examining the immune system of pigs, we will better understand how they deal with and eliminate pathogens and how the adaptive immune system can be supported.

These investigations are also important regarding zoonotic diseases such as influenza (23, 24). Pig endemic viruses represent a serious threat to public health (25). This aspect is especially relevant in the context of the worldwide pork industry. Along with poultry, pork is the most consumed meat in the world. In 2023, worldwide pork production reached an impressive total of more than 120 million metric tons. For 2022, the pork market was valued at 254 billion US$ and is expected to grow by 2028, from 8% to 418 billion US$. In 2022, China took the lead in global pork production, with more than 55 million metric tons of pork, accompanying 452 million pigs of livestock. Closely, the European Union secured the second position, while the United States claimed the third place [published by M. Shahbandeh, Apr 13, 2023, https://www.statista.com/statistics/273232/net-pork-production-worldwide-by-country/]. Considering the large number of pigs worldwide, it is highly important to avoid mass culling to prevent the spread of highly contagious diseases such as influenza, African swine fever or foot-and-mouth disease, to reduce and avoid spillover events. These aspects are even more critical due to the global trade and transportation of meat products around the world.

In this study, we investigated for the first time the N-glycosylation of IgG during the ontogenesis of pigs. For the characterization of N-glycosylation status, we used several analytical approaches and demonstrated that distinct changes in IgG glycosylation occur over time.

## 2 Materials and methods

### 2.1 Animals and sample collection

Animal samples for this project originated from purebred German Landrace pigs. The housing conditions are described in detail in the publication of Murani (26). Sample collection from the animals took place through rapid anterior vena cava puncture at 08:00 am; this procedure involved one-week-old piglets (7 days), one day prior to weaning (26 days), and one day following weaning (28 days). Upon reaching a body weight of approximately 105 kg (at the age of 150 days, peripubertal age), trunk blood was collected during exsanguination at the time of slaughter within the experimental slaughter facility at FBN, Dummerstorf. For this study, plasma samples from six individuals with an equal distribution of male and female piglets were used. Male piglets underwent castration at four days of life. The plasma was prepared by centrifugation for 20 min at 4°C and 2,000 × g and stored at -80°C until use.

### 2.2 Chemicals

All chemicals and reagents were of analytical grade. Double-distilled water was obtained from an in-house Milli-Q system. The acetonitrile (ACN) (Honeywell Riedel-de Haën) and methanol (Roth) used were of HPLC grade. Solvents used for mass spectrometric analysis: Dichlormethan (DCM), water and ACN were purchased from Merck and were of GC-MS and LC-MS grade, respectively.

### 2.3 IgG isolation

To remove the remaining cellular components, the plasma was centrifuged at 13,000 rpm for 20 min. Then, 50 µl of plasma was diluted with 200 µl of PBS (pH 7.4) and applied to a 1 ml Sepapure protein A column (Knauer). After the column was equilibrated with PBS (pH 7.4), the remaining plasma components were washed off using PBS as washing buffer. The bound IgG were eluted with 0.1 M Glycin (Roth) (pH 3.0) and subsequently neutralized with 1 M Tris (pH 9). To remove salt, the samples were dialyzed overnight at 4°C against 50 mM NH_4_HCO_3_. After this, the samples were lyophilized. The dried IgG samples were resolved in PBS (pH 7.4), and the protein concentration was measured using a Pierce Micro BCA Protein Assay Kit (Thermo Fisher Scientific) according to the manufacturer’s instructions.

### 2.4 SDS-PAGE

The isolated IgG fractions were investigated by sodium dodecyl sulfate-polyacrylamide gel electrophoresis (SDS-PAGE). Two micrograms of protein from each sample was diluted in reducing sample buffer (Thermo Fisher Scientific) and denatured for 5 min at 95°C. The separation was performed on a 10% gel. To visualize the proteins, the gel was incubated in a staining solution containing 20% Roti Blue (Roth) and 20% methanol overnight. To destain the background, the gel was transferred to a 25% methanol solution.

### 2.5 Quantification of neutral monosaccharides by GC-MS

The reduction of monosaccharides and subsequent acetylation are two of the most suitable methods for analyzing complex hydrolysates (27, 28) without needing further purification (29). The applied method was adapted from Brunton et al. (30). To this end, the samples were hydrolyzed in 500 µl of 4 N TFA for 4 h at 100°C. After hydrolyzation samples were reduced with 500 µl 1% NaBH_4_ for 12 h at room temperature, neutralized with acetic acid, dried and acetylated with 400 µl acetic anhydride in 100 µl pyridine by incubation for 12 h at room temperature in the dark. Acetylated samples were extracted with DCM/H_2_O and the organic phase was used for GC-MS analysis. After drying all samples were resolved in 100 µl of DCM.

The analysis of the processed monosaccharides was performed with an Agilent Technologies GC-MS system (7890A/5975C). Acetylated monosaccharides were separated on a HP-5MS column with Helium as carrier gas and a constant flow of 1 ml/min. The temperature was set to 260°C for the inlet with a septum purge flow of 3 ml/min and a purge flow to split vent of 50 ml/min at 0.5 min. The oven temperature was set up according to the following gradient in **Table 1**.

**Table 1:** GC-MS gradient.

Rate [°C/min]	Temperature [°C]	Hold Time [min]	Run Time [min]
	80	1	1
10	180	10	21
3	290	0	57.667

For detection, the selected ion monitoring mode was used with a resolution set to 1.5 and an electron multiplier voltage of 600. A mixture of monosaccharides, including Fuc, Man, glucose (Glc), Gal, GlcNAc and N-acetylgalactosamine (GalNAc), was used to determine the retention time and for quantification. Xylose (Xyl) was added as an internal standard to verify the chemical reactions and extraction steps. Fuc was detected with mass/charge (*m/z*) ratios of 217 and 231, whereas for Xyl 145 and 217, for Hexoses 217 and 289 and for the N-acetylhexosamines 144 and 360 were used.

### 2.6 Sialic acid quantification by HPLC

For the quantification of sialic acids, Neu5Ac and N-glycolylneuraminic acid (Neu5Gc) were released under acidic conditions and labeled with the fluorophore 1,2-diamino-4,5-methylene dioxybenzene (DMB) (Dojindo Laboratoriese) (31). To release sialic acids, the samples were dissolved in 0.2 M TFA (Roth) and incubated at 80°C for 4 h. For DMB labeling, the samples were dried and subsequently resolved in 80 μl of DMB reaction buffer (9 mM sodium hydrosulfite, 1 M beta-mercaptoethanol, 20 mM TFA and 2.7 mM DMB). The labeling occurred at 55°C for 2 h. The reaction was stopped by adding 20 μl of 0.2 N NaOH. Different concentrations of Neu5Ac and Neu5G standards (Sigma-Aldrich) were used for quantification. All chemical reactions occurred under excess of reagents to ensure a complete hydrolyzation and labeling. The resulting DMB-labeled sialic acids were analyzed by reverse phase HPLC using a Superspher 100 C-18 column (250 mm × 40 mm, Merck-Hitachi) at 40°C column temperature. The eluents used were E1 with methanol, acetonitrile and water (4:4:92 with 0.1% TFA) and E2 with methanol, acetonitrile and water (45:45:10 with 0.1% TFA) with a flow rate of 0.25 ml/min and the following gradient: 0 min - 100% E1; 2 min - 100% E1; 25 min - 95% E1; 31 min - 0% E1; 40 min - 0% E1; 41 min - 100% E1; and 55 min - 100% E1. The excitation and emission wavelengths were 372 nm and 456 nm, respectively.

### 2.7 Analysis of N-glycans

The N-glycans were enzymatically released under denaturing conditions by PNGase F (New England Biolabs) according to the manufacturer’s instructions. In brief, the dried IgG was diluted in glycoprotein denaturing buffer, heated for 10 min at 100°C and subsequently cooled on ice. The remaining components for the reaction, glycobuffer 2 and 1% NP-40 in addition to PNGase F, were added and incubated at 37°C for 2 h. Afterwards, the N-glycans were separated from the proteins by Chromabond C18 columns (Macherey-Nagel). The columns were equilibrated before use with methanol, 0.1% TFA, 40% ACN, 80% ACN, 0.1% TFA and ddH_2_O. The samples were loaded on the column and washed with 0.1% TFA. The flow through included the N-glycans and was collected and dried in a SpeedVac.

To detect the N-glycans, the samples were labeled with the fluorophore procainamide hydrochloride (Abcam). To this end, the dried samples were dissolved by 3 min of sonication in 100 µl of label buffer containing 70 µl of dimethyl sulfoxide (DMSO) (Roth), 30 µl of glacial acetic acid (Merck), 10 mg of procainamide hydrochloride and 6 mg of NaBH_3_CN (Sigma-Aldrich). Fluorescence labeling was performed at 65°C for 3 h (32). To remove excess procainamide hydrochloride from the reaction, the samples were dialyzed overnight at 4°C against water and subsequently dried in a SpeedVac.

The analysis of N-glycans was performed by hydrophilic interaction liquid chromatography (HILIC) HPLC using an Accucore-150-Amide-HILIC column (100 × 2.1 mm, 2.6 µm particle size) and an Accucore-150-Amide-HILIC (10 × 2.1 mm, 2.6 µm particle size) guard column (both from Thermo Fisher Scientific). The following mobile phases were used: Eluent E1: 80% acetonitrile with 50 mM ammonium formate (VWR Chemicals); and E2: 50 mM ammonium formate, pH 4.5. The gradient was as follow: 0 min - 100% E1; 15 min - 77% E1; 30 min - 68% E1; 30.5 min - 100% E1; 40 min - 100% E1. The flow rate was 0.5 ml/min, and the column temperature was 60°C. Signal fluorescence detection (FD) occurred at wavelengths of 305 nm for excitation and 360 nm for emission. To determine the N-glycans within the peaks at a given retention time, peaks were collected, dried and subjected to LC-MS analysis. The procainamide hydrochloride-labeled glucose homopolymer ladder dextran (Ludger) was used for reproducibility checks. For the relative quantification of agalactosyalted, galactosylated and sialylated N-glyans the peak-ratios of these groups are compared within a sample. An absolute quantification is not applicable, since it cannot be excluded that the enzymatic release of N-glycans has taken place completely.

The fractionated N-glycans were additionally analyzed via ultra performance liquid chromatography and heated electrospray ionization mass spectrometry (UPLC-HESI-MS(/MS)). Chromatography was performed on an UPLC Vanquish (Thermo Scientific) with the same column and guard column used for fluorescence detection. The mobile phases used were as follows: Eluent 1 (E1): 90% ACN, 10 mM ammonium formate, 0.1% HCOOH; E2 = 50% ACN, 10 mM ammonium formate, 0.1% HCOOH. The gradient used for separation was 0 min - 65% E1; 27 min - 20% E1; 28 min - 5% E1; 33 min - 5% E1; 33.1 min - 65% E1; 50 min - 65% E1, the flow rate was 0.5 ml/min, and the column temperature was 45°C. The N-glycans were detected by a Qexactive plus mass spectrometer (Thermo Scientific) with HESI ionization operating in positive and negative ionization mode. The mass range was set to *m/z* 300-2800 with a resolution for full MS of 70000 and for data dependent MS/MS of 17500.

### 2.8 Ethical statement

The animal samples used within this study were approved by The Animal Care Committee of the Research Institute for Farm Animal Biology and the State Mecklenburg-Western Pomerania (Landesamt für Landwirtschaft, Lebensmittelsicherheit und Fischerei; LALLF M-V/TSD/7221.3-2.1-010/12) (26). Sample collection, animal care and handling were in accordance with the German Law of Animal Protection.

### 2.9 Statistics

The statistical analysis were performed using the GraphPad Prism software (version 10.2.0). Due to the repeated measurement of the individuals, repeated measures ANOVA was applied to analyze the animal-to-animal variability. The time points were compared pairwise with each other by using Tukey test. Significant differences were defined as follows: * p < 0.1, ** p < 0.01, *** p < 0.001, and **** p < 0.0001.

## 3 Results and discussion

### 3.1 IgG isolation and verification of purity

**Figure 2:**
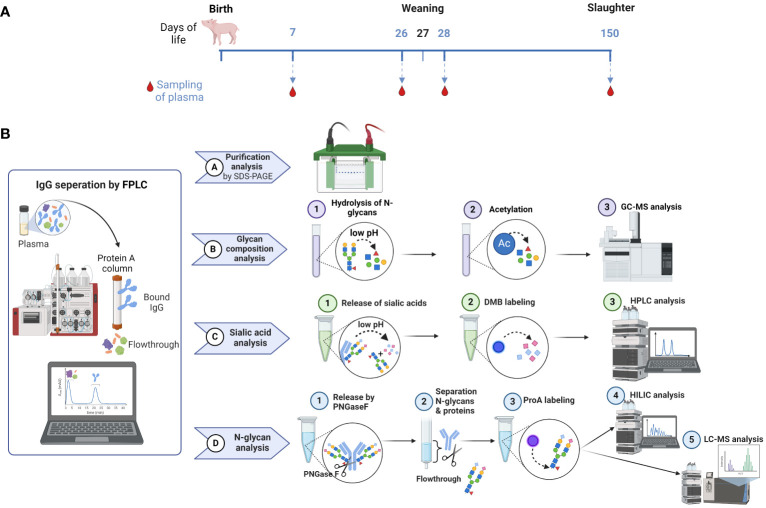
Overview of sample collection and workflow. **(A)** Sample collection occurred on day 7 after birth, one day before (26) and one day after (28) weaning, as well as on the day of slaughtering on day 150. **(B)** The workflow displays the isolation of IgG from plasma by FPLC and the subsequent steps, including the verification of the IgG isolation by SDS-PAGE, the analysis of the glycan composition by GC-MS, the sialic acid quantification by RP-FD and the fluorescent analysis of N-glycans by HILIC-FD and HILIC-HESI-MS(/MS). Man, mannose; Gal, galactose; GlcNAc, N-Acetylglucosamine; Fuc, fucose; Neu5Ac, N-acetylneuraminic acid. Created with BioRender.com.

In this study, we investigated the N-glycans of IgGs and their distribution in pigs over a time period from the age of seven days to 150 days. These time points are relevant stages of pork production and can be divided into following stages: suckling, weaner, grower and finisher stages. During the suckling period, piglet losses of up to 14% can occur in the first few weeks after birth (33). Some of these losses are due to infections, like pathogens causing diarrhea, leading to increased mortality and therapeutic cost (34, 35). That makes it to the first critical phase of life. The next critical time point after birth and suckling is weaning at the age of three to five weeks, depending on the farming system (conventional vs. organic). Weaning is likely the most stressful event in the life of a pig, accompanied by challenges to the digestive and immune systems, which can lead to problems such as poor health, feed intake and growth (36). For this reason, we were interested in the changes that accompany this event and collected blood samples at the ages of 26 and 28 days. The sampled pigs were held in a conventional system where they were weaned at the age of 27 days. The third stage, the finisher stage, is the last stage in a pig’s life and ends when the pigs achieve their slaughter weight. These conditions are typically reached at approximately 150 to 160 days of age (5-6 months). This shows that the ages of 26, 28 and 150 days are highly important for pig farming. **Figure 2A** illustrates the sample collection plan according to the associated pig age. Moreover, the analytical strategies used are displayed in **Figure 2B**.

**Figure 3:**
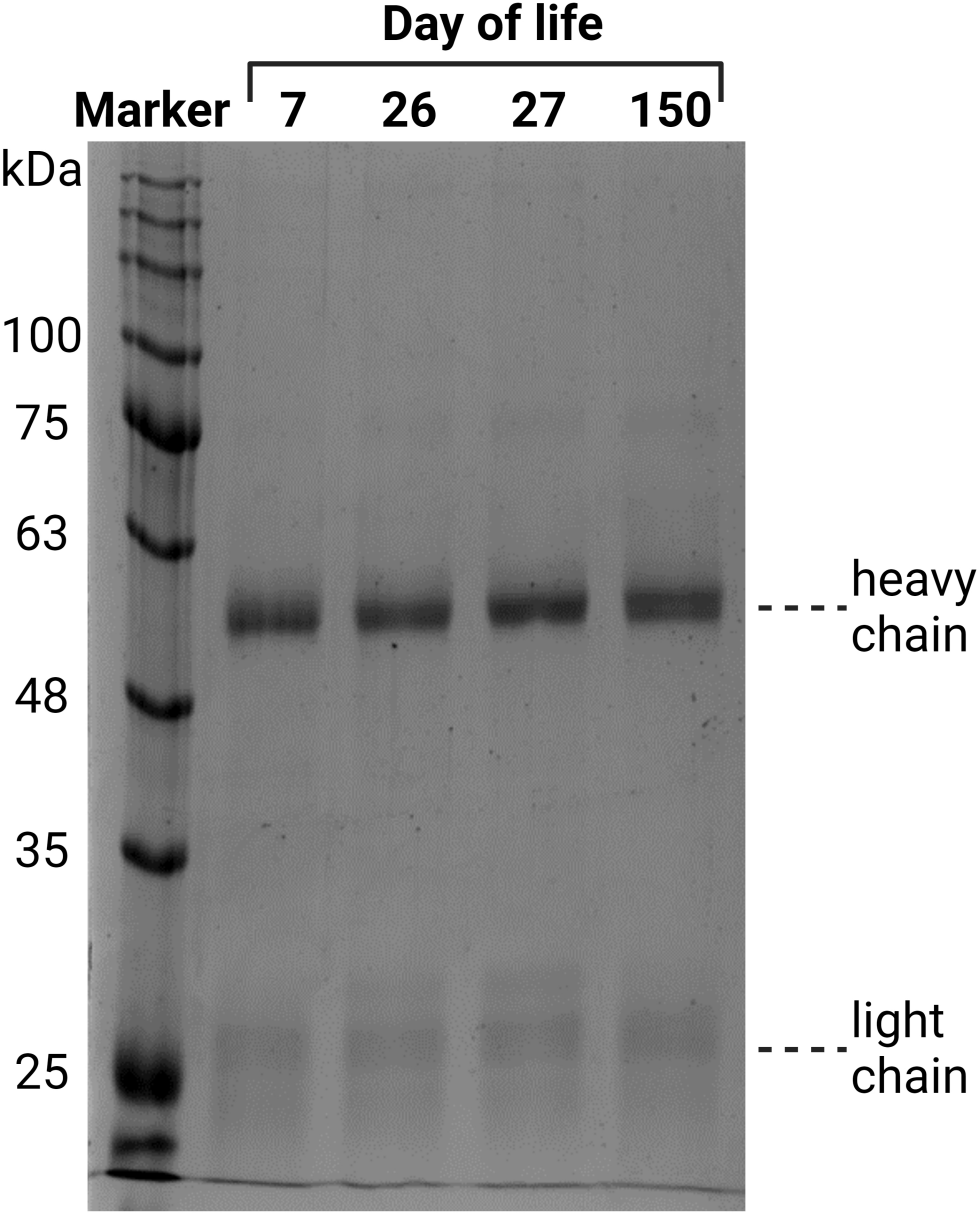
Verification of the purity of the isolated IgG from plasma, exemplary for one animal from all four time points. The isolated IgGs were separated by denaturing SDS-PAGE. Two micrograms of protein were separated and stained with Coomassie blue.

First, IgGs were isolated from blood plasma using a protein A column. The eluates were dried, after which the protein content was determined. To evaluate the purity of the isolated IgG, 2 µg of each sample was analyzed via SDS-PAGE (**Figure 3**). Proteins were visualized by Coomassie blue staining. Since the disulfide bonds of IgG were reduced by DTT, the heavy chain at 50 kDa and the light chain at 25 kDa were visualized. A light shadow was observed at 75 kDa, which might be the result of an incomplete reduction in IgG (37). The signal intensities were comparable in the analyzed samples. The obtained results verify that this isolation technique is reliable and that IgG is the main purified protein.

### 3.2 Monosaccharide composition analysis of N-glycans

**Figure 4:**
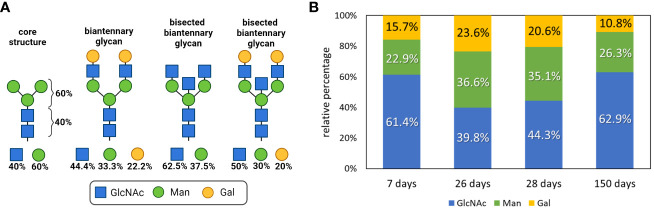
Glycan composition analysis during postnatal development. **(A)** N-glycan structures of IgGs and the respective proportions of the monosaccharides are shown. Created with BioRender.com. **(B)** Neutral monosaccharides were analyzed by GC-MS after hydrolysis and peracetylation. The proportions of GlcNAc (N-Acetylglucosamine), Man (mannose) and Gal (galactose) are displayed. Analyses that are more detailed are shown in **Supplementary Figure 3**.

The composition of an N-glycan can be calculated by determining the ratios of GlcNAc to Man to Gal. The core structure of all N-glycans comprises two GlcNAc residues and three Man residues, leading to the following composition: 40% GlcNAc and 60% Man (**Figure 4A**). With increasing elongation of monosaccharides to form complex N-glycans, the proportion of Man decreases, whereas the GlcNAc level increases. For instance, a bisected biantennary glycan with two galactosylated GlcNAc branches linked to the core structure contains five GlcNAc, three Man and two Gal residues, so that the ratios change as follows: 30% Man, 50% GlcNAc and 20% Gal. However, since IgGs contain several different N-glycans, the examined values are an average of all present N-glycan structures. Consequently, only a trend can be displayed.

To determine the amount of the building blocks of the N-glycans on the isolated IgG samples, the N-glycans were hydrolyzed, and the resulting neutral monosaccharides were reduced and peracetylated for GC-MS analysis. The results are shown in **Figure 4B**. We observed that within the first postnatal weeks, from day 7 to 26, the amounts of Man and Gal increase from 22.9 to 36.6% and from 15.7 to 23.6%, respectively. However, the level of GlcNAc decreased from 61.4 to 39.8%. In 26-day-old pigs, the N-glycans of IgG consisted of an average of four GlcNAc, three Man and two Gal residues (**Figure 4**). Remarkably, until the age of 150 days, the value for Gal decreased and the proportion of GlcNAc increased. These results suggest that fewer GlcNAc residues are elongated with Gal on day 150 than at younger ages. During the first 4 weeks, the isolated IgGs originated mainly from the milk because the piglets do not produce significant amounts of endogenous IgG at this age. In contrast, at the age of five months, pigs produce IgG themselves. Based on only these obtained values, the endogenous IgG of 150-day-old pigs was mainly decorated with small N-glycans, which induce predominant pro-inflammatory responses, whereas the maternal IgGs that were received contained more anti-inflammatory structures. However, the examined data are only the first trend and need to be verified with additional experiments, such as the quantification of sialic acids.

### 3.3 Sialic acid quantification

**Figure 5:**
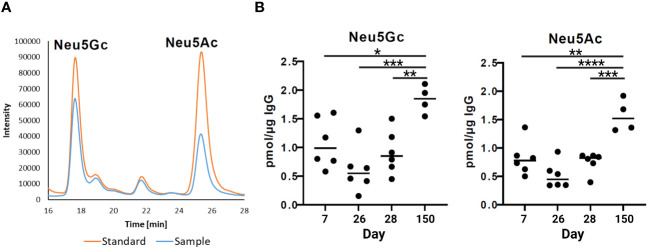
Sialylation status of IgGs during postnatal development. **(A)** Representative chromatograms of a standard containing fluorescently labelled Neu5Gc (N-glycolylneuraminic acid) and Neu5Ac (N-acetylneuraminic acid) and a sample are shown. **(B)** Analysis of sialic acids present on IgG by RP-chromatography. The amounts of Neu5Gc and Neu5Ac per 1 µg of IgG are shown. For the statistical analysis, mixed effect analysis and multiple comparison Tukey test were applied. Significant differences are given: * p < 0.1, ** p < 0.01, *** p < 0.001, and **** p < 0.0001.

The strongest anti-inflammatory effects have sialylated N-glycans. The N-glycans at the IgG Fc can be elongated by one sialic acid residue per galactosylated antenna. In pigs, two different kinds of sialic acids can be attached, Neu5Ac and Neu5Gc (38). To determine the sialylation status of porcine IgG during development, the amounts of the sialic acids Neu5Ac and Neu5Gc in the isolated IgG fractions were analyzed via RP-HPLC (**Figure 5A**). To this end, sialic acids were released from IgG by hydrolysis and labeled with the fluorophore DMB. Interestingly, the levels of Neu5Ac and Neu5Gc were comparable, and the amount of both sialic acids increased with age (**Figure 5B**). At the age of seven days, the levels of Neu5Ac and Neu5Gc were approximately 1 pmol/µg IgG and decreased to 0.5 pmol/µg IgG on day 26. No significant changes occurred between days 26 and 28. Thus, no rapid effects were induced by weaning. However, thereafter, sialylation significantly increased until day 150, when it reached 1.6 pmol/µg IgG in the case of Neu5Ac and 1.8 pmol/µg IgG in the case of Neu5Gc. This corresponds to a threefold increase from day 26 to day 150. Thus, at the age of five months, the N-glycans of endogenous IgG contain higher amounts of sialic acid than the N-glycans on day 7, which originate primarily from maternal IgGs. This finding is in contrast to the results from the neutral monosaccharide analysis, which demonstrated that the ratio of Gal, the needed acceptor for a transfer of sialic acid residue, decreased.

### 3.4 Changes in N-glycan distribution during development

**Figure 6:**
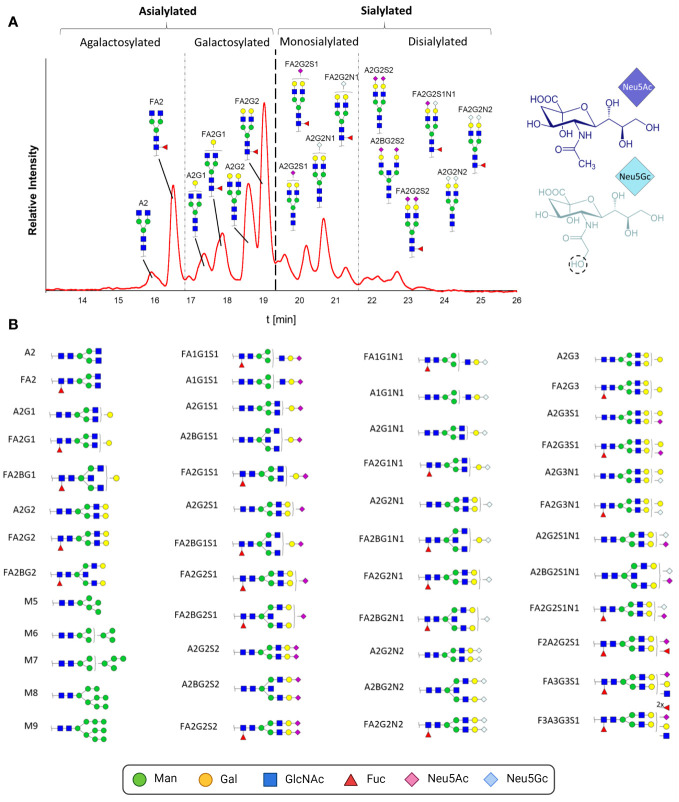
N-glycan analysis of IgGs during postnatal development. **(A)** Fluorescently labelled N-glycans were separated by HILIC. A representative chromatogram from one animal at the age of 26 days is displayed. The peaks detected during the HILIC-FD analysis were fractionated and analyzed by UPLC-HESI-MS(/MS), and the identified glycans were assigned. The division of the chromatogram into a, mono- and disialylated structures confirmed the different retention times of the N-glycans. The chemical structures of Neu5Ac and Neu5Gc are also displayed. **(B)** All detected and assumed N-glycan structures based on the UPLC-HESI-MS(/MS)analysis. To depict the glycans, the Symbol Nomenclature For Glycans (SNFG) was used, and for their description, the Oxford Notation was selected. Man, mannose; Gal, galactose; GlcNAc, N-acetylglucosamine; Fuc, fucose; Neu5Ac, N-acetylneuraminic acid; Neu5Gc, N-glycolylneuraminic acid. Corresponding MS-data can be found in **Supplementary Figure 1**.

To resolve this alleged contradiction between the glycan composition analysis and sialylation status, we characterized the structure of the intact N-glycans and determined the ratios of the present pro- and anti-inflammatory N-glycans. For this purpose, N-glycans were enzymatically released by PNGaseF, fluorescently labeled and analyzed by HILIC. This technique is suitable for glycan separation, whereby under the conditions used, less polar glycans elute earlier than glycans with higher polarity. In the case of N-glycans, this means that smaller structures, e.g., paucimannose, elute first. The retention time increases with the branching and length of the antennas in addition to the number of sialic acid residues. In **Figure 6A**, the chromatographic separation of the fluorescently labeled N-glycans of one IgG sample is displayed. To assign the N-glycan structures to the signals, the peaks were fractionated and subsequently analyzed by LC-MS. A total of 48 different N-glycan structures were identified (**Figure 6B**; corresponding MS data in **Supplementary Figure 1**). The complex N-glycans include both fucosylated and biantennary structures. In line with the results of the sialic acid analysis, the N-glycans contained not only Neu5Ac but also Neu5Gc. The presence of two different sialic acids in pigs, Neu5Ac and Neu5Gc, leads to IgG N-glycan structures, which are more heterogenic than those in humans. The difference between these two sialic acids is the additional hydroxyl group in Neu5Gc at the acetylamino group (**Figure 6A**). The conversion of Neu5Ac to Neu5Gc is catalyzed by the enzyme cytidine monophosphate-N-acetylneuraminic acid hydroxylase (CMAH). In humans, the CMAH gene contains a frameshift mutation. As a consequence, the enzyme cannot be produced (39). Because the inactive enzyme Neu5Gc is absent in humans (40), but Neu5Gc still can be recognized and incorporated into human glycan structures by sialyltransferases originating from the diet (41). Neu5Gc has been described in humans in the context of pathological disorders (42, 43), such as in association with cancer development or xenograft-related problems (40). Apart from the interest in the prevention of Neu5Gc within the production of therapeutics, including immunoglobulins and donor organs for humans as medical therapies, the biological function of Neu5Gc on IgG in mammals naturally occurring this sialic acid has not been investigated so far.

Most of the detected N-glycans were also described in previous studies analyzing porcine IgG (37, 38, 40, 44–46). The group of Buist detected a total of 16 different glycans (44). Only two of them were not fucosylated, and three of the 16 were not sialylated; thus, all 13 sialylated structures were also fucosylated. Lopez and colleagues observed only seven different N-glycans (37). The detected sialylated structures had only one Neu5Gc, and no Neu5Ac was observed. Nelson and colleagues detected a total of 12 glycans, including sialylated structures, which were terminated only by Neu5Ac residues. The highest heterogeneity was observed by Burlak’s group (40). In total, 37 different N-glycans, including comparably sialylated structures, were identified. The differences in sialylation status between the mentioned studies could be due to lower detection limits and/or harsher processing and MS techniques, which can lead to the loss of unstable sialic acid residues. For instance, it is known that MALDI-TOF-MS analysis of sialylated N-glycans, which was commonly used in previous studies, can lead to the release of sialic acid residues during ionization (47, 48). Moreover, breed, age or nutrition might influence the sialylation status of N-glycans.

In addition to complex N-glycans, traces of high-mannose structures, which are uncommon in human IgG, were detected. The origin of these glycans might be from parallel isolated IgM. It is described that protein A is not exclusively specific for IgG and binds also poorly to IgM [www.thermofisher.com, (49, 50)]. It is therefore possible that the detected high-mannose structures originate from IgM, as human IgM also contains high-mannose structures (1). The Fab region of the isolated IgG might be an additional source of high-mannose structures since approximately 15 to 25% of all human IgGs also contain N-glycans on this side, whereby these glycosylation sites are not conserved (14). There are only few studies on Fab glycosylation, but Bond and colleagues described the presence of high-mannose structures in the Fab region of human IgG (51).

**Figure 7:**
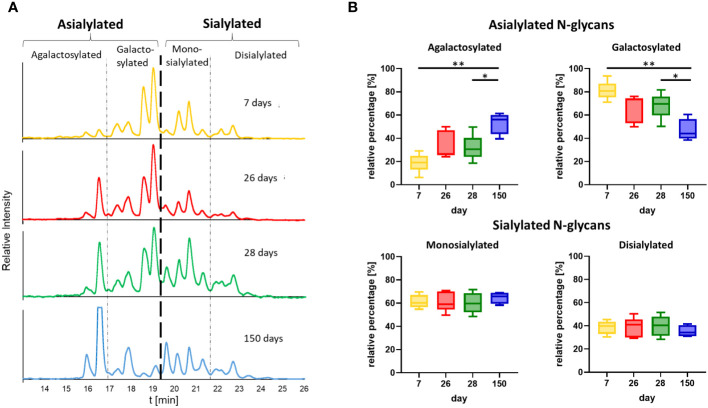
Time point comparison of N-glycan composition. **(A)** Representative chromatograms of the N-glycans from one animal at all four time points are shown. The chromatograms are divided into peaks corresponding to asialylated and sialylated N-glycans. The asialylated N-glycans are additionally divided into agalactosylated and galactosylated N-glycans and the sialylated in mono- and disialylated N-glycans. The chromatogram at the age of 28 days corresponds to the chromatogram in **Figure 6A**. The chromatograms of all further animals can be found in **Supplementary Figure 2**. **(B)** The peak areas of the agalactosylated, galactosylated, mono- and disialylated structures (please see **Supplementary Figure 2** for the chromatograms) were determined and the ratios of the asialylated (agalactosylated/galactosylated) and sialylated (monosialylated/disialylated) N-glycans were calculated respectively. Box & Whisker plots (median; min to max) are shown. For the statistical analysis, mixed effect analysis and multiple comparison Tukey test were applied. Significant differences are given: * p < 0.1, ** p < 0.01.

On the basis of the MS analyses of the collected peaks, it was possible to assign the peaks to the corresponding structures, confirming that shorter and asialylated glycans elute earlier in HILIC than longer and sialylated structures (**Figure 6A**). Monosialylated glycans have shorter retention times than structures with two sialic acid residues. Furthermore, structures with Neu5Gc elute later than those with Neu5Ac. This is due to the additional hydroxyl group of Neu5Gc (**Figure 6B**), which endows the molecule with more hydrophilic properties. The results demonstrated that the asialylated N-glycans eluted between 15 and 19 min, the monosialylated N-glycans eluted mainly between 19 and 21.5 min, and the disialylated N-glycans eluted after 21.5 min (**Figure 6A**). Using these analytical strategies, the N-glycan pattern of IgG at each individual time point was examined to obtain an overall picture of the dynamic IgG glycosylation during aging (**Figure 7**). In 7-day-old piglets, predominantly N-glycans with two galactosylated GlcNAc branches linked to the core structure (A2G2 and FA2G2) in addition to their monosialylated forms were observed. Interestingly, up to the time of weaning, the amount of shorter agalactosylated N-glycans increased (F2A). A change in glycosylation in the direct context of weaning could not be observed. Presumably, the time span of two days (day 26 vs 28) is too short for this to occur. In contrast, remarkable alterations occurred during the following weeks. In 150-day-old pigs, the amount agalactosylated N-glycans continued to increase (A2 and F2A). Together with sialylated N-glycans they represent the dominating N-glycan structures, whereas the amount of asialylated galactosylated N-glycans dramatically decreases. This contrasting trend explains the alleged contradiction between the increase in sialylation and the decrease in Gal during aging.

As mentioned in the introduction, the glycosylation status impacts the effector functions of IgG. In general, it influence the binding of the IgG-Fc domain to Fc receptors (1, 52). The IgG Fc fragment has the shape of a horseshoe and can have an open or closed conformation, depending on the glycosylation (53, 54). For example, an non-glycosylated human IgG-Fc has a closed conformation preventing interaction and binding to Type I Fc receptors, which includes FcγRI, FcγRIIa,b,c, FcγRIIIa and b (55). This closed conformation instead enable the binding to Type II FcR including CD23 and DC-SIGN (55). Further, glycans such as FA2 lead to an open, but FA2G2S2 to an closed conformation (12). How and to what extent Neu5Gc influences the conformation is still unclear and requires further investigation. In addition, corefucosylation is known to reduce ADCC. Interestingly, sialylation reduces ADCC as well but only, if the N-glycans are corefucosylated. Based on these studies, our results suggest that, in postnatal piglets, mainly glycans are present which predominantly induce a mild pro-inflammatory response, since mainly galactosylated structures are present. In contrast, in 150-day-old pigs, the IgG pool changed to a balanced ratio of strongly pro- and strongly anti-inflammatory glycosylation, with less galactosylated structures. At the age of seven days, piglets have solely absorbed milk IgGs, whereas at the age of 150 days, pigs produce endogenous IgG. Thus, the observed changes in glycosylation might be a result of the maturation of the innate immune system and an optimization of the inflammatory system to the existing environment, including repeated contact with specific pathogens.

## 4 Conclusion

How glycosylation changes with age in pigs has not been determined so far. These results indicate that with the maturation of the innate immune system, remarkable changes occur in the glycosylation status and thus in the activity of porcine IgGs. However, functional studies are needed to explain the dynamics that drive this process and how the glycosylation of IgG can be used to positively influence the course of an infectious disease. These findings could also contribute to optimizing the passive immunization of piglets via milk to prevent disease, and the described results are the necessary basis for such novel developments.

## Data availability statement

The original contributions presented in the study are included in the article/**Supplementary Material**. Further inquiries can be directed to the corresponding author.

## Ethics statement

The animal study was approved by The Animal Care Committee of the Research Institute for Farm Animal Biology and the State Mecklenburg-Western Pomerania (Landesamt für Landwirtschaft, Lebensmittelsicherheit und Fischerei; LALLF M-V/TSD/7221.3-2.1-010/12). The study was conducted in accordance with the local legislation and institutional requirements.

## Author contributions

KZ: Conceptualization, Data curation, Formal analysis, Funding acquisition, Investigation, Methodology, Project administration, Resources, Software, Supervision, Validation, Visualization, Writing – original draft, Writing – review & editing. LI: Investigation, Methodology, Writing – review & editing. CEG: Methodology, Writing – review & editing. EM: Resources, Writing – review & editing. SPG: Conceptualization, Funding acquisition, Validation, Writing – original draft, Writing – review & editing.
