# Supplementary material for: Changes in the N-glycosylation of porcine immune globulin G during postnatal development
Source: Front Immunol. 2024 Apr 18;15:1361240. doi: 10.3389/fimmu.2024.1361240 (PMC11063267; doi:10.3389/fimmu.2024.1361240)
Supplement: Supplementary file 1 [file DataSheet_1.pdf]

Supplementary Material

1 Supplementary Data

1.1 Supplementary Figures

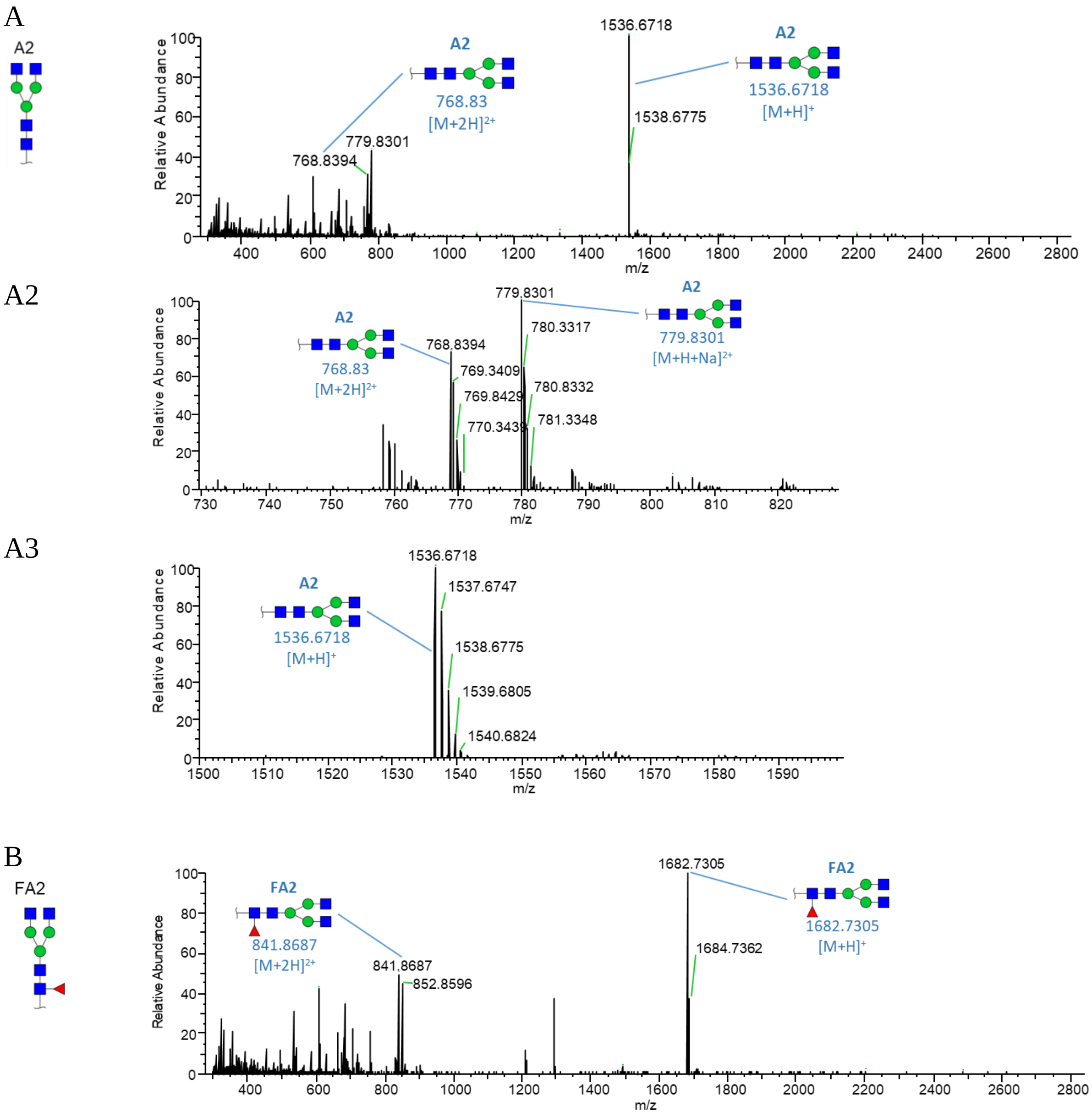

B2

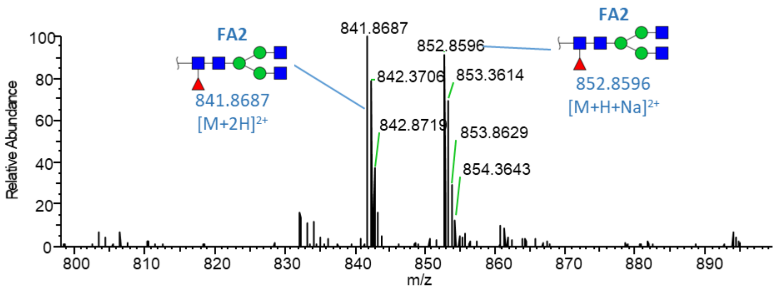
FA2
841.8687
[M+2H]2+
FA2
852.8596
[M+H+Na]2+
841.8687
842.3706
842.8719
852.8596
853.3614
853.8629
854.3643
Relative Abundance
m/z

B3

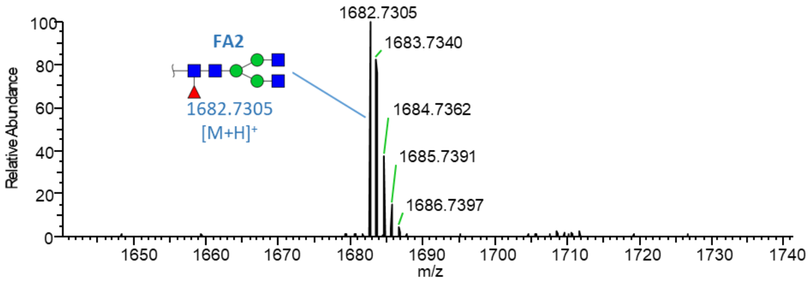
FA2
1682.7305
[M+H]+
1682.7305
1683.7340
1684.7362
1685.7391
1686.7397
Relative Abundance
m/z

C

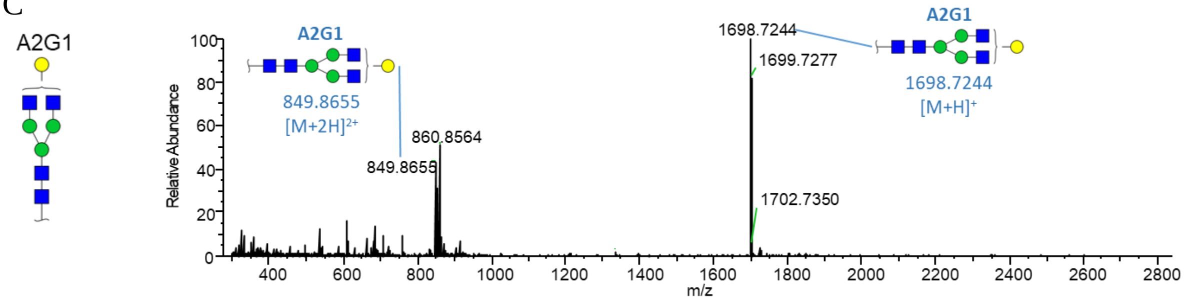
A2G1
A2G1
849.8655
[M+2H]2+
A2G1
1698.7244
[M+H]+
1698.7244
1699.7277
860.8564
849.8655
1702.7350
Relative Abundance
m/z

C2

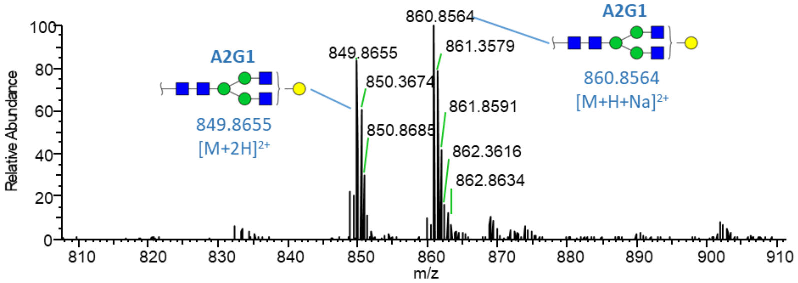
A2G1
849.8655
[M+2H]2+
A2G1
860.8564
[M+H+Na]2+
860.8564
861.3579
849.8655
850.3674
861.8591
850.8685
862.3616
862.8634
Relative Abundance
m/z

C3

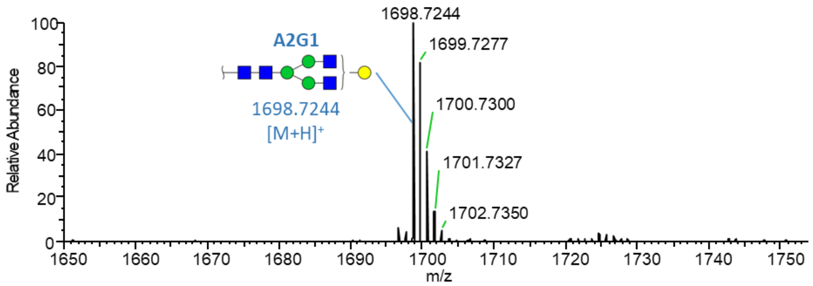
A2G1
1698.7244
[M+H]+
1698.7244
1699.7277
1700.7300
1701.7327
1702.7350
Relative Abundance
m/z

D

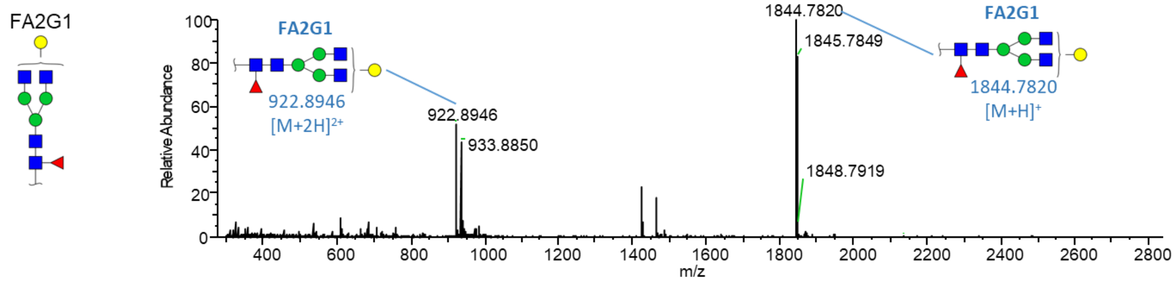
FA2G1
Relative Abundance
m/z
FA2G1
922.8946
[M+2H]2+
922.8946
933.8850
1844.7820
1845.7849
1848.7919
FA2G1
1844.7820
[M+H]+

D2

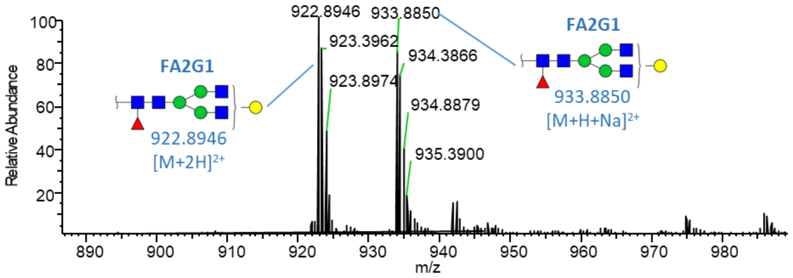
Relative Abundance
m/z
922.8946
923.3962
923.8974
933.8850
934.3866
934.8879
935.3900
FA2G1
922.8946
[M+2H]2+
FA2G1
933.8850
[M+H+Na]2+

D3

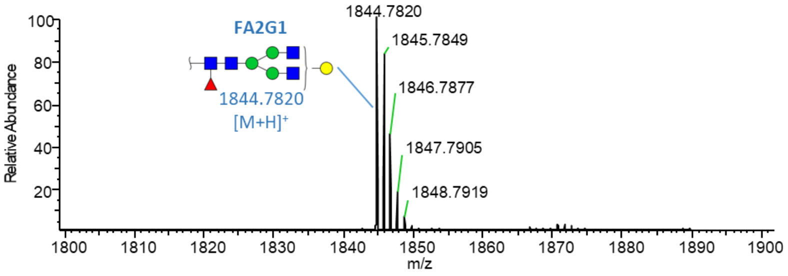
Relative Abundance
m/z
FA2G1
1844.7820
[M+H]+
1844.7820
1845.7849
1846.7877
1847.7905
1848.7919

E

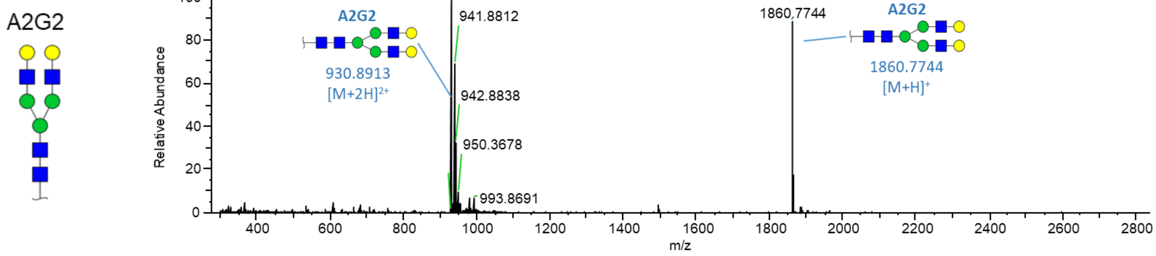
A2G2
Relative Abundance
m/z
A2G2
930.8913
[M+2H]2+
930.8913
941.8812
942.8838
950.3678
993.8691
1860.7744
A2G2
1860.7744
[M+H]+

E2

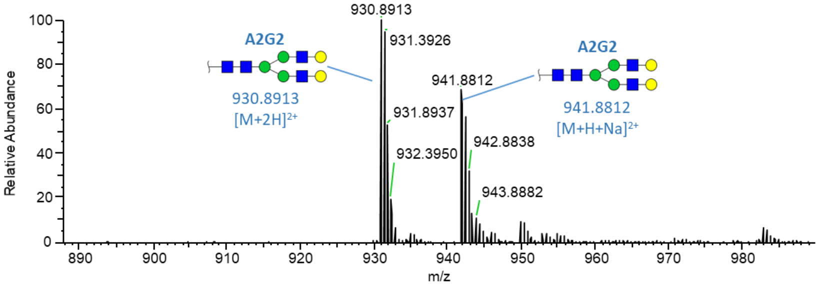
Relative Abundance
m/z
A2G2
930.8913
[M+2H]2+
930.8913
931.3926
931.8937
932.3950
941.8812
942.8838
943.8882
A2G2
941.8812
[M+H+Na]2+

E3

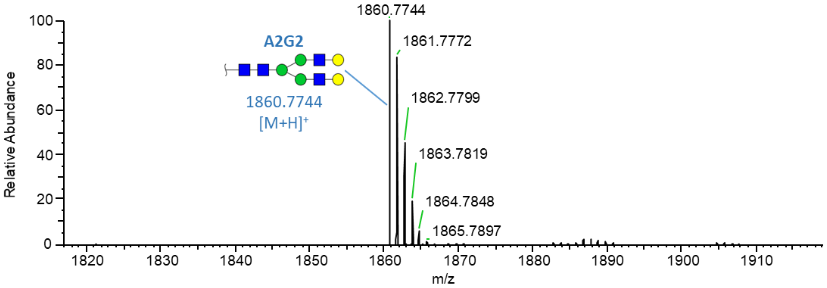

F

FA2G2

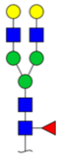

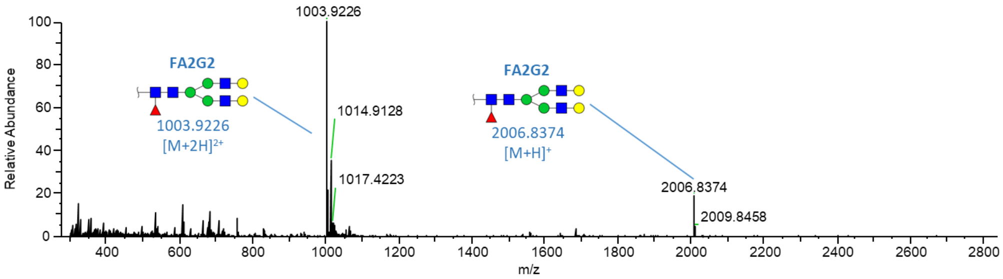

F2

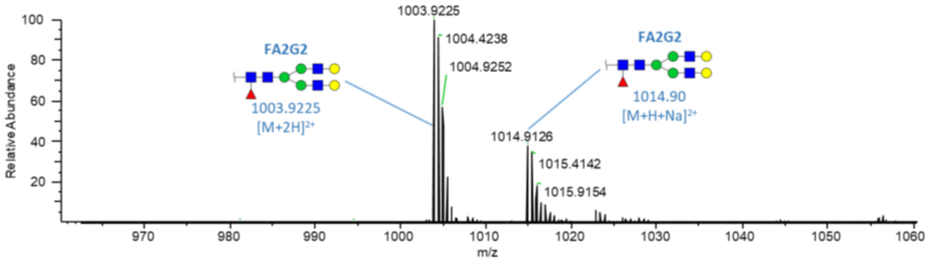

G

A2G2S1

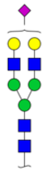

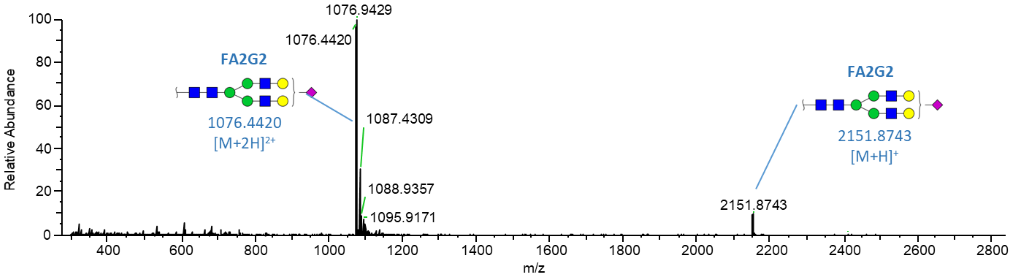

G2

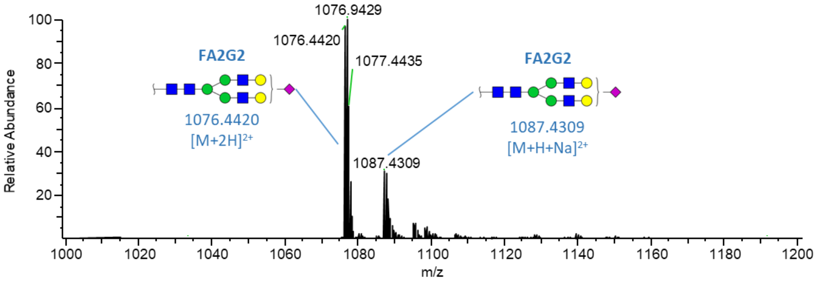

H

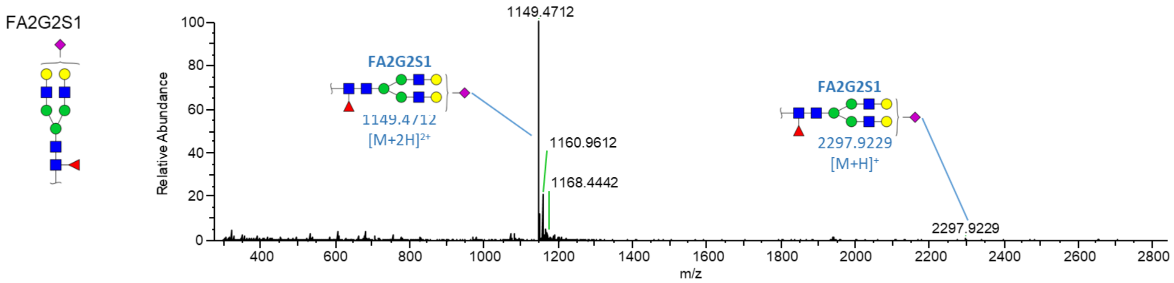
FA2G2S1
1149.4712
FA2G2S1
1149.4712
[M+2H]2+
1160.9612
1168.4442
FA2G2S1
2297.9229
[M+H]+
2297.9229
Relative Abundance
m/z

H2

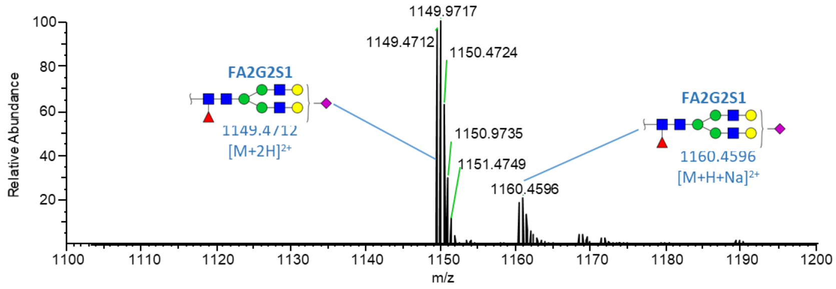
1149.9717
1149.4712
1150.4724
FA2G2S1
1149.4712
[M+2H]2+
1150.9735
1151.4749
1160.4596
FA2G2S1
1160.4596
[M+H+Na]2+
Relative Abundance
m/z

I

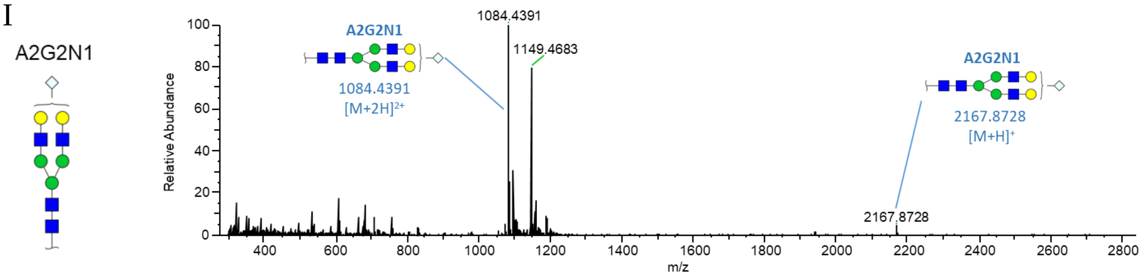
A2G2N1
1084.4391
A2G2N1
1084.4391
[M+2H]2+
1149.4683
A2G2N1
2167.8728
[M+H]+
2167.8728
Relative Abundance
m/z

I2

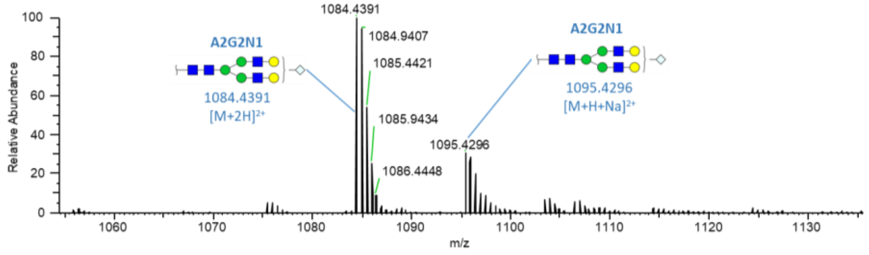
1084.4391
A2G2N1
1084.4391
[M+2H]2+
1084.9407
1085.4421
1085.9434
1086.4448
A2G2N1
1095.4296
[M+H+Na]2+
1095.4296
Relative Abundance
m/z

J

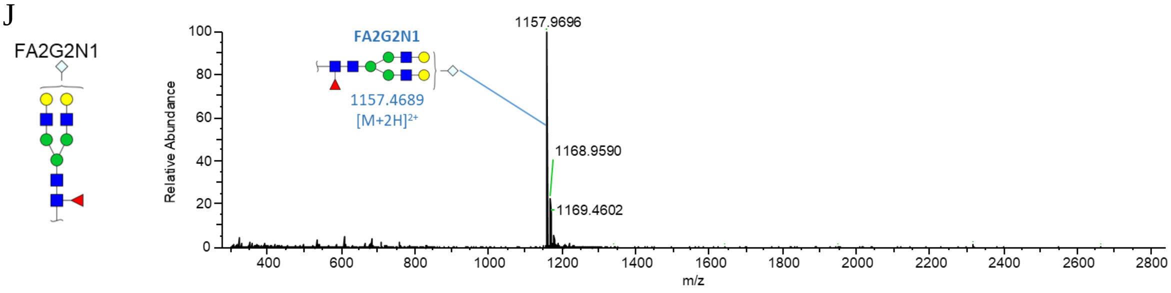
FA2G2N1
1157.9696
FA2G2N1
1157.4689
[M+2H]2+
1168.9590
1169.4602
Relative Abundance
m/z

J2

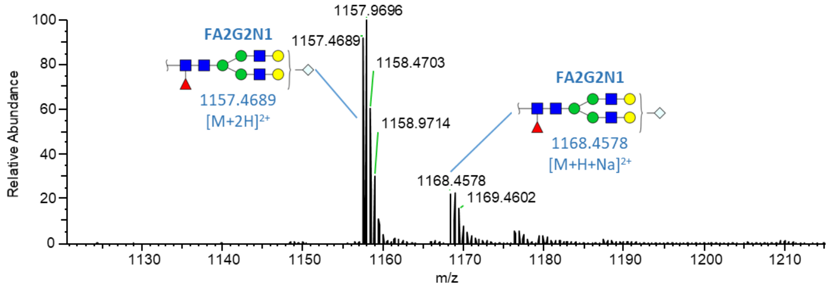
1157.9696
1157.4689
FA2G2N1
1157.4689
[M+2H]2+
1158.4703
1158.9714
FA2G2N1
1168.4578
[M+H+Na]2+
1168.4578
1169.4602
Relative Abundance
m/z

K

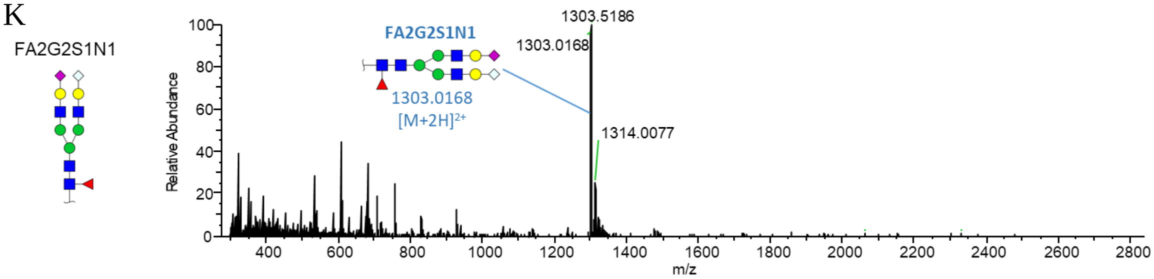
FA2G2S1N1
FA2G2S1N1
1303.0168
[M+2H]2+
1303.5186
1303.0168
1314.0077
Relative Abundance
m/z

K2

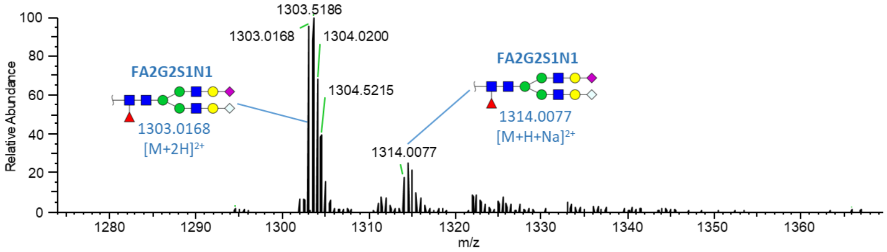
1303.5186
1303.0168
1304.0200
1304.5215
FA2G2S1N1
1303.0168
[M+2H]2+
FA2G2S1N1
1314.0077
[M+H+Na]2+
1314.0077
Relative Abundance
m/z

Supplementary Figure 1 Representative MS spectra for the most abundant glycans. The glycans are named by the Symbol Nomenclature For Glycans (SNFG). The *m/z* data are indicated for the different charged glycans with Procainamide A and are depicted separately.

1-x

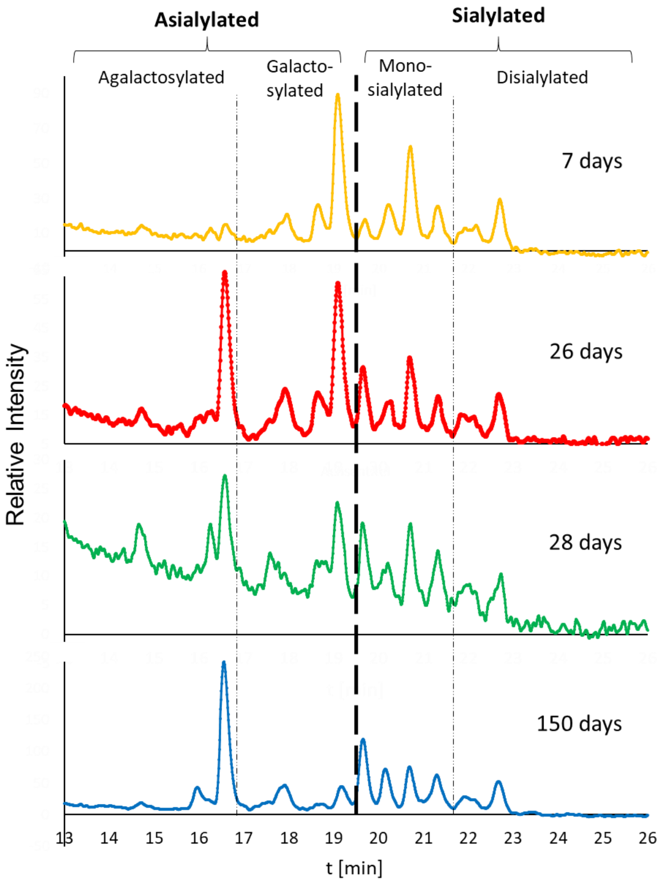
Asialylated
Sialylated
Agalactosylated
Galacto-
sylated
Mono-
sialylated
Disialylated
7 days
26 days
28 days
150 days
Relative Intensity
13
14
15
16
17
18
19
20
21
22
23
24
25
26
t [min]

2-x

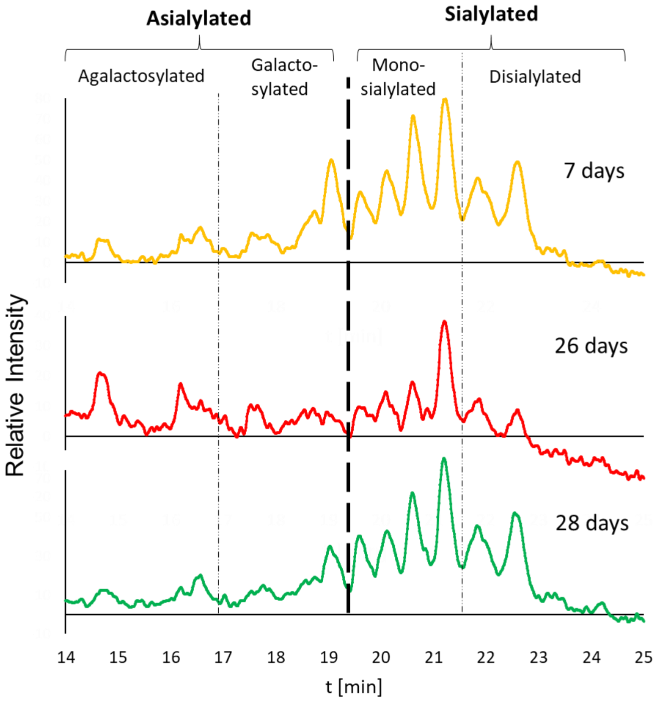
Asialylated
Sialylated
Agalactosylated
Galacto-
sylated
Mono-
sialylated
Disialylated
7 days
26 days
28 days
Relative Intensity
14
15
16
17
18
19
20
21
22
23
24
25
t [min]

3-x

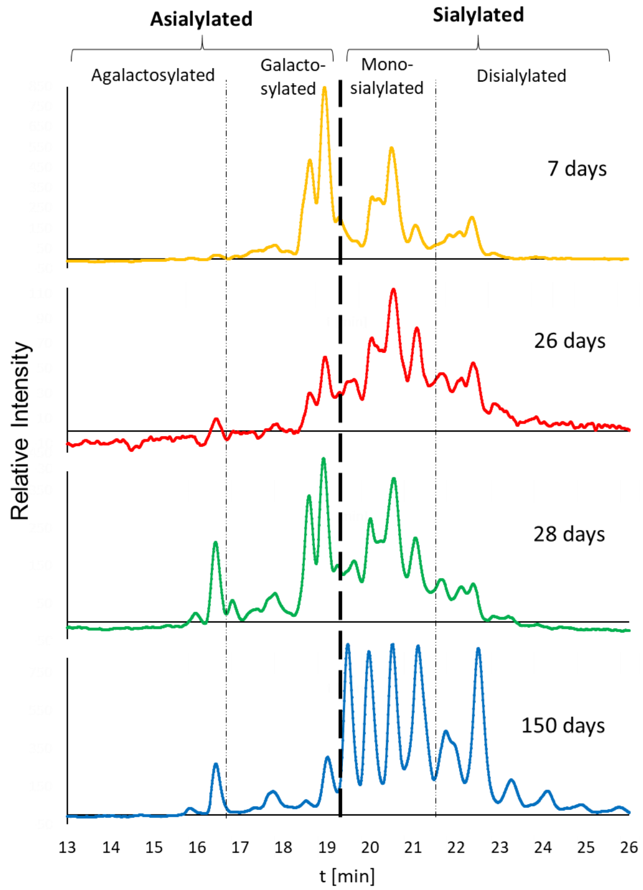

Asialylated
Sialylated
Agalactosylated
Galacto-sylated
Mono-sialylated
Disialylated
7 days
26 days
28 days
150 days
Relative Intensity
13 14 15 16 17 18 19 20 21 22 23 24 25 26
t [min]

4-x

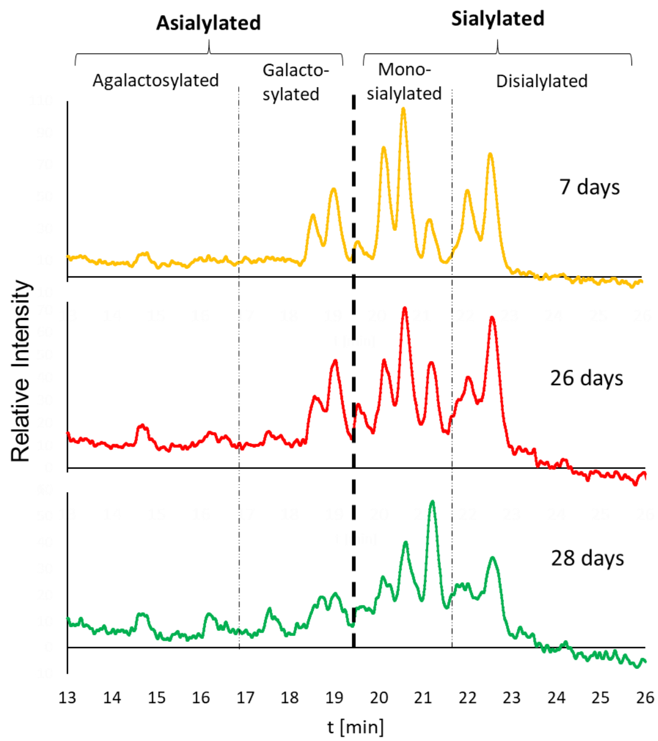

Asialylated
Sialylated
Agalactosylated
Galacto-sylated
Mono-sialylated
Disialylated
7 days
26 days
28 days
Relative Intensity
13 14 15 16 17 18 19 20 21 22 23 24 25 26
t [min]

6-x

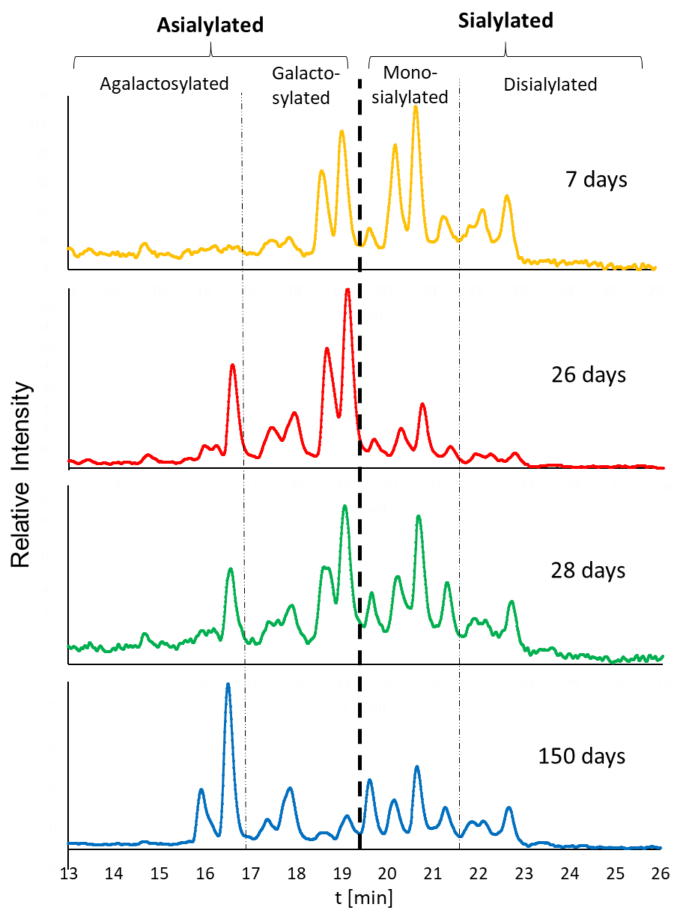

Supplementary Figure 2 N-glycan analysis during postnatal development. Fluorescently labelled N-glycans were analyzed by HILIC. Chromatograms of 5 different animals are shown. The chromatograms are divided into peaks corresponding to a-, mono- or disialylated N-glycans. The asialylated N-glycans are additionally divided into agalactosylated and galactosylated N-glycans. For animals 2 and 4 there is no final time point of 150 days.

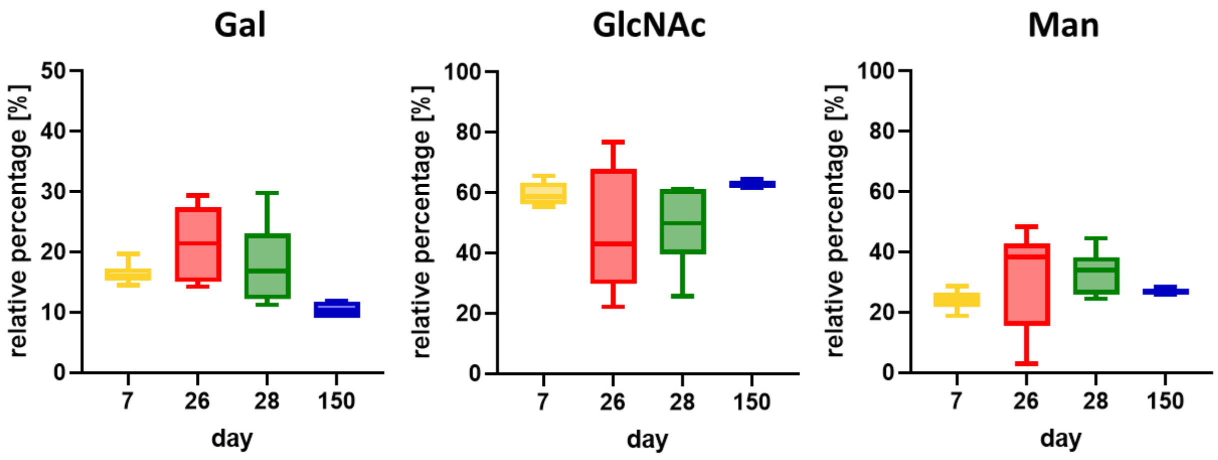

Supplementary Figure 3 Glycan composition analysis of IgGs during postnatal development. As described in Figure 4, neutral monosaccharides were analyzed by GC-MS after hydrolysis and peracetylation. Box & Whisker plots (median; min to max) are shown for the calculated monosaccharide ratios.

1.2 Supplementary Tables

Supplementary Table 1 Calculated *m/z*. Listed are the calculated *m/z* for N-glycans from Supplementary Figure 1. Shown are the Ion masses of different charged glycans with and without Procainamide A.

			**Singly positive charged**	**Doubly positive charged**	
		Ion	$[M+H]^+$	$[M+2H]^{2+}$	$[M+H+Na]^{2+}$
Procainamide A	219.155	Mass of	1.01	2×1.01	1.01+22.99
			**Calculated *m/z***		
	**A2**	1316.49	1317.49	659.25	670.24
+ Procainamide A	**A2**	1535.64	1536.65	768.83	779.82
	**FA2**	1462.54	1463.55	732.28	743.27
+ Procainamide A	**FA2**	1681.70	1682.71	841.86	852.85
	**FA2G1**	1624.60	1625.60	813.31	824.30
+ Procainamide A	**FA2G1**	1843.75	1844.76	922.88	933.87
	**A2G1**	1478.54	1479.55	740.28	751.27
+ Procainamide A	**A2G1**	1697.69	1698.70	849.85	860.85
	**A2G2**	1640.59	1641.60	821.30	832.29
+ Procainamide A	**A2G2**	1859.75	1860.75	930.88	941.87
	**FA2G2**	1786.65	1787.66	894.33	905.32
+ Procainamide A	**FA2G2**	2005.81	2006.81	1003.91	1014.90
	**A2G2S1**	1931.69	1932.69	966.85	977.84
+ Procainamide A	**A2G2S1**	2150.84	2151.85	1076.43	1087.42
	**FA2G2S1**	2077.75	2078.75	1039.88	1050.87
+ Procainamide A	**FA2G2S1**	2296.91	2297.91	1149.46	1160.45
	**A2G2N1**	1947.68	1948.69	974.85	985.84
+ Procainamide A	**A2G2N1**	2166.84	2167.84	1084.43	1095.42
	**FA2G2N1**	2093.74	2094.75	1047.88	1058.87
+ Procainamide A	**FA2G2N1**	2312.90	2313.90	1157.46	116845
	**FA2G2S1N1**	2384.84	2385.84	1193.43	1204.42
+ Procainamide A	**FA2G2S1N1**	2603.99	2605.00	1303.00	1313.99
